# Artificial intelligence literacy, anxiety, and attitudes among registered nurses across different hospital tiers: a multi-center cross-sectional study

**DOI:** 10.3389/fpubh.2026.1880523

**Published:** 2026-08-12

**Authors:** Yingqi Dai, Xili Zhao, Xiao Pan, Jiaxin Wei

**Affiliations:** 1 The Second Affiliated Hospital of Chongqing Medical University, Chongqing, China; 2 Acute and Critical Care Nursing Innovation and Translation Engineering Research Center of Chongqing Education Commission of China, Chongqing, China; 3 Chongqing Geriatric Specialized Nursing Clinical Diagnosis and Treatment Center, Chongqing, China

**Keywords:** anxiety, artificial intelligence, attitudes, literacy, registered nurses

## Abstract

**Background:**

The rapid advancement of artificial intelligence is driving an unprecedented technological transformation in nursing. However, the successful integration of these technologies depends largely on the proficiency and perspectives of registered nurses. Consequently, there is an urgent need to examine the psychological and behavioral responses of this workforce.

**Methods:**

A multi-center, cross-sectional survey was conducted from March to May 2026. a stratified convenience sampling method was employed to recruit 1,392 registered nurses from tertiary hospitals, secondary hospitals, and community health centers in Chongqing. Data collection instruments included a general demographic questionnaire, the Artificial Intelligence Literacy Scale, the Artificial Intelligence Anxiety Scale, and the General Attitudes Towards Artificial Intelligence Scale. Statistical analyses, including descriptive statistics, Spearman correlation, and multiple linear regression.

**Results:**

A total of 1,392 registered nurses participated in the study, with a mean age of 34.69 ± 7.13 years. AI literacy scored 5.27 ± 0.90 (75.29% scoring rate). AI anxiety was moderate (51.29%), with the highest concerns appearing in socio-technical blindness (56.43%) and job replacement (55.14%). Overall, nurses maintained a positive attitude toward AI (74.00%). AI literacy was negatively correlated with anxiety (*r* = −0.338, *p <* 0.001) and significantly positively correlated with attitude (*r* = 0.551, *p* < 0.001), anxiety and attitude were negatively correlated (*r* = −0.541, *p* < 0.001). Regression analysis indicated that AI literacy was a significant associated factor for both anxiety levels (*B* = -0.453, *p* < 0.001) and attitudes (*B* = 0.377, *p* < 0.001), explaining 9.9 and 29.4% of the variance, respectively. Additionally, a lower frequency of electronic device use (*p* = 0.024) and lack of proficiency in device operation (*p* = 0.033) were associated with higher anxiety levels, whereas male nurses demonstrated more positive attitudes compared to female nurses (*p* = 0.011).

**Conclusion:**

Nurses demonstrated high AI literacy and positive attitudes; however, anxiety remained prominent. Enhancing AI literacy may alleviate psychological anxiety, with device accessibility and usage patterns also playing critical roles. To facilitate the effective integration of artificial intelligence into clinical practice, administrators should strengthen institutional support mechanisms alongside providing facility resources and conventional education, thereby promoting the full utilization and translation of available resources.

## 1 Introduction

Artificial Intelligence (AI), as the core force of contemporary technological change, is defined as “software or hardware systems designed by humans that, given a complex goal, perceive their environment through data acquisition, interpret collected structured or unstructured data (1), reason on knowledge or process information derived from this data, and decide on the best actions to achieve the specified goal (2).” AI encompasses various technologies, including machine learning (ML), deep learning (DL), and natural language processing (NLP) (3). In the 1950s, Christopher Strachey developed the first AI program. Today, AI technology is integrating into numerous fields, including healthcare, education, and transportation (4). With continuous technological evolution, the comprehensive penetration of AI into the medical field is driving an unprecedented technological transformation (5). AI technology is fundamentally changing the way medical services are delivered, from disease diagnosis and treatment selection to patient monitoring, greatly enhancing the accuracy and efficiency of healthcare services (6). By solving complex clinical problems and optimizing resource allocation, AI plays a pivotal role in improving patient outcomes and overall quality of care (7). While healthcare professionals benefit from these advancements, they also face the challenge of directly applying data-driven insights from the real world to patient care to achieve efficient, high-quality care and large-scale transformation (8), and healthcare providers must possess the necessary competencies to better adopt these technologies in clinical practice (9).

As the medical field undergoes overall digitalization, the delivery of nursing services is also experiencing a major shift. AI technology has permeated various stages, such as real-time monitoring of vital signs, intelligent alerts for fall risks, automated generation of clinical nursing pathways, and natural language processing for nursing documentation (10). As healthcare systems increasingly face challenges—such as growing patient demand, rising complexity in care requirements, resource constraints, and developmental imbalances—AI serves as a promising solution to boost efficiency and improve patient outcomes (11). Throughout this technology-driven transition, the group of registered nurses has demonstrated irreplaceable core value. As the largest professional force in the healthcare system and those who interact most frequently with patients, registered nurses are the direct implementers of all intelligent devices and clinical decisions (12). Nevertheless, a potential mismatch often exists between their technical readiness and actual competence, which may undermine the clinical efficacy of AI and compromise care quality (13). The individual traits of registered nurses significantly influence the integration of AI technology into clinical practice, and the successful application of AI in clinical settings relies on a deep understanding of nurses as the end-user group. At the same time, assessing nurses’ current level of AI knowledge is crucial, as it helps clarify future training needs (14). AI literacy encompasses not only basic operational skills but also deep cognitive understanding and ethical decision-making capabilities (15). High levels of AI literacy are believed to improve the quality of patient care (16). However, the intervention of new technology often comes with technological anxiety. Nurses commonly worry about keeping pace with technological developments and face anxiety regarding learning curves and potential job displacement; such negative emotions can significantly impact their professional self-efficacy (17–20). Research by Özçevik Subaşi et al. (21) indicates that nurses’ attitudes toward AI significantly influence their literacy and anxiety levels. Positive attitudes toward AI have a notable positive effect on nurses’ innovative work behavior (22). A systematic review of existing literature reveals that current research in this field is limited by narrow study populations and mostly descriptive accounts of single dimensions. Furthermore, there is a lack of large-scale survey research in this area. Against this backdrop, this study is theoretically grounded in the Technology Acceptance Model (TAM) (23) and the expanded TAM 3.0 model by Venkatesh and Bala (24). Classic TAM theory posits that an individual’s external characteristics, such as technological literacy, serve as critical antecedents influencing their subjective psychological perceptions and attitudes. Concurrently, the TAM 3.0 extension model indicates that technological anxiety essentially represents a negative emotional manifestation or a barrier index reflecting perceived ease of use. Therefore, this study incorporates artificial intelligence literacy as an external characteristic variable and introduces artificial intelligence anxiety as a psychological perceptual indicator that mirrors barriers to perceived ease of use. This study aims to investigate the current status of artificial intelligence literacy, anxiety, and attitudes among registered nurses, analyze their influencing factors, and explore the impact of registered nurses’ artificial intelligence literacy on artificial intelligence anxiety and technological attitudes, thereby providing a targeted basis for digital interventions to better implement technology in clinical settings.

## 2 Method

### 2.1 Study design

This study was a multicenter, cross-sectional survey utilizing a stratified convenience sampling method to conduct a questionnaire-based investigation in Chongqing. Registered nurses from 10 tertiary hospitals, 6 secondary hospitals, and 14 community hospitals in Chongqing were selected as the study population between March and May 2026. The inclusion criteria were: (1) registered nurses; (2) working independently in clinical frontline positions for 6 months or longer; (3) providing informed consent and participating voluntarily in this study. The exclusion criteria were: (1) non-clinical frontline nurses, including administrative staff, logistics personnel, and retired personnel rehired; (2) individuals continuously away from clinical work for 6 months or longer due to sick leave, maternity leave, or external training; (3) interns or short-term rotating trainees; (4) individuals whose returned questionnaires were of substandard quality, characterized by an extremely short completion time, missing critical core variables, or obvious logical contradictions. This study was reported in accordance with the STROBE (Strengthening the Reporting of Observational Studies in Epidemiology) guidelines (25).

### 2.2 Sample size

The minimum sample size was estimated using the rule of thumb, which suggests a sample size of 5, 10, or 20 times the number of variables (26). A sample size 20 times the number of variables satisfies the most stringent application of this rule. Given that 21 variables were analyzed in this study, a baseline sample size of 420 participants was required. Accounting for an estimated 20% non-response or invalid questionnaire rate, the minimum target sample size was set to 504 cases.

### 2.3 Instruments

#### 2.3.1 General demographic questionnaire

A self-designed questionnaire was used to collect demographic data, including gender, age, educational background, years of professional experience, hospital tier, professional title, availability of AI-related equipment in the department, prior AI training, frequency of AI training, proficiency in using electronic devices, and frequency of electronic device use at work.

#### 2.3.2 Artificial Intelligence Literacy Scale (AILS)

The AILS, developed by Wang et al. (27), targets the general population within the context of human-AI interaction. It consists of 12 items across four dimensions: awareness, usage, evaluation, and ethics. Items are rated on a 7-point Likert scale, ranging from 1 (“Strongly Disagree”) to 7 (“Strongly Agree”). Total scores range from 12 to 84, with higher scores indicating higher AI literacy. The original scale reported a Cronbach’s alpha of 0.83. In this study, the Cronbach’s alpha was 0.809, the *KMO* value was 0.915, and the Bartlett’s test of sphericity was significant (*p* < 0.001).

#### 2.3.3 Artificial Intelligence Anxiety Scale (AIAS)

Developed by Wang et al. (28), this scale comprises 21 items categorized into four dimensions: learning, job replacement, socio-technical blindness, and AI configuration. Items are rated on a 7-point Likert scale (1 = “Strongly Disagree” to 7 = “Strongly Agree”). Scores range from 21 to 147, where higher scores reflect greater AI-related anxiety. The original AIAS demonstrated a Cronbach’s alpha of 0.964. In the present study, the Cronbach’s alpha was 0.977, the *KMO* value was 0.971, and the Bartlett’s test of sphericity was significant (*p* < 0.001).

#### 2.3.4 General Attitudes Toward Artificial Intelligence Scale (GAAIS)

Originally developed by Schepman et al. (29), this scale includes 20 items divided into positive (items 1–12) and negative (items 13–20) dimensions. The original Cronbach’s alpha was 0.88 for the positive dimension and 0.83 for the negative dimension. Items are rated on a 5-point Likert scale (1 = “Strongly Disagree” to 5 = “Strongly Agree”). Negative items are reverse-scored; once reversed, higher total GAAIS scores indicate more positive attitudes. The Chinese version, adapted by Huang et al., retains 15 items (30), seven of which are reverse-scored. The Chinese GAAIS reported a Cronbach’s alpha of 0.875, with 0.833 and 0.865 for the positive and negative dimensions, respectively. In this study, the overall Cronbach’s alpha was 0.865, with the positive and negative dimensions yielding 0.928 and 0.927, respectively.

### 2.4 Ethical issues and data quality control

This study protocol was approved by the Ethics Committee of the Second Affiliated Hospital of Chongqing Medical University prior to the survey (Approval No: 2026EC148) and was conducted in accordance with the Declaration of Helsinki. Participation was voluntary, anonymous, and confidential, with no personally identifiable information collected. An electronic informed consent form was provided on the landing page of the questionnaire, detailing the research objectives and the principles of data confidentiality. All participants were required to read and actively check the informed consent option before starting the questionnaire. The survey was administered to registered nurses in Chongqing via an online platform and distributed through WeChat and other social media channels. Prior to sending the links, we communicated with the administrators or department nurse managers of the target hospitals to explain the purpose of the survey and obtain their permission. Subsequently, the investigation period and the coordinators for each site were determined. A standardized completion guide was provided via the WeChat platform, which explicitly detailed how to respond to the questionnaire. The guide and links were distributed by the project supervisor to the coordinators at the target hospitals, who then forwarded them through department work groups for completion. To prevent duplicate submissions, the survey settings restricted access to a single entry per IP address. To ensure data quality, the research team conducted a pre-test prior to formal deployment to verify questionnaire logic and establish baseline completion metrics. The average completion time under conditions of rapid, logical responding was determined to be 120 s; consequently, 120 s was established as the completion time cutoff value for a valid questionnaire. An immediacy check for completeness was performed on all returned questionnaires. A total of 1,433 questionnaires were retrieved. After excluding those that failed to meet the quality requirements—including a completion time of less than 120 s, missing or incorrect entries, and obvious logical errors—1,392 valid questionnaires remained, yielding an effective response rate of 97%.

### 2.5 Data analysis

Statistical analyses were performed using SPSS 27.0, with the significance level set at *α* = 0.05 (*p* < 0.05 indicating statistical significance). Frequencies and percentages were used to describe general demographic data. Scores for the literacy, anxiety, and attitude scales—along with their respective dimensions—were reported as mean ± standard deviation. Additionally, the scoring rate for each dimension was calculated to standardize the scales for comparison. The Kolmogorov–Smirnov (*K-S*) test was employed to assess data normality. As the data were not normally distributed, they were described using medians (maximum, minimum) and interquartile ranges, and non-parametric tests were used for intergroup comparisons. Specifically, the Mann–Whitney *U* test was used for comparisons between two groups, while the Kruskal–Wallis *H* test was applied for comparisons among multiple groups. Spearman correlation analysis was conducted to examine the relationships between AI literacy, anxiety, and attitudes. Given that multiple linear regression primarily relies on the normality of residuals, preliminary assumption testing and model diagnostics were performed prior to conducting the regression analysis. Residual histograms and normal *P–P* plots were utilized to verify the normal distribution of residuals. Homoscedasticity was assessed and model diagnostics were conducted by plotting scatterplots of the standardized residuals against the standardized predicted values. Multicollinearity was evaluated using the variance inflation factor (*VIF*). The effect sizes for all regression analyses are reported as unstandardized coefficients (*B*) along with their corresponding 95% confidence intervals (95% *CI*).

## 3 Results

### 3.1 Demographic characteristics

A total of 1,392 registered nurses were included in this study, consisting of 182 from community health centers, 299 from secondary hospitals, and 911 from tertiary hospitals. The sample was predominantly female (*n* = 1,340, 96.3%), with males accounting for 3.7% (*n* = 52). Regarding professional development, 458 participants (32.9%) had received AI-related training; however, only 36 of those (7.9%) attended more than five training sessions per year on average. The mean age of the participants was 34.69 ± 7.13 years. Detailed participant characteristics are presented in Table 1.

**Table 1:** Demographic characteristics.

Variable	*N* (%)
Gender	
Male	52 (3.7)
Female	1,340 (96.3)
Age	
≤30	410 (29.5)
31–40	753 (54.1)
>40	229 (16.5)
Education	
Secondary specialized school	12 (0.9)
Junior college	239 (17.2)
Bachelor’s degree	1,125 (80.8)
Master’s degree or higher	16 (1.1)
Years of experience	
≤10 years	592 (42.5)
11–20 years	610 (43.8)
>20 years	190 (13.6)
Hospital tier	
Community hospital	182 (13.1)
Regional hospital	299 (21.5)
Tertiary care hospital	911 (65.4)
Professional title	
Nurse	163 (11.7)
Senior Nurse	447 (32.1)
Nurse-in-Charge	668 (48.0)
Associate Chief Nurse	105 (7.5)
Chief Nurse	9 (0.6)
AI equipment availability	
Yes	296 (21.3)
No	1,096 (78.7)
Prior AI training	
Yes	458 (32.9)
No	934 (67.1)
Frequency of AI training (per year)^a^	
≤3	300 (65.5)
4–5	122 (26.6)
>5	36 (7.9)
Device proficiency	
Very proficient	516 (37.1)
Generally proficient	824 (59.2)
Not proficient	52 (3.7)
Frequency of electronic device use	
Often	350 (25.1)
Occasionally	675 (48.5)
Very few	367 (26.4)
Age	
Mean ± SD (min-max)	34.69 ± 7.13 (20–59)

*N* = 1,329.

^a^Percentages for “Frequency of AI training” were calculated based on the subsample of participants who had received prior AI training (*n* = 458).

### 3.2 Status of AI literacy, anxiety, and attitude among registered nurses

The survey results indicate that the total AI literacy score for registered nurses was 5.27 ± 0.90 (scoring rate: 75.29%). The scoring rates for each dimension, ranked from highest to lowest, were: Evaluation (77.14%), Awareness (76.71%), Usage (73.57%), and Ethics (73.29%). The total AI anxiety score was 3.59 ± 0.57 (scoring rate: 51.29%). Across dimensions, Socio-technical blindness anxiety showed the highest scoring rate (56.43%), followed by job replacement anxiety (55.14%), while AI configuration anxiety (51.43%) and learning anxiety (45.71%) were relatively lower. The total score for AI attitude was 3.70 ± 0.65 (scoring rate: 74.00%). Notably, the scoring rate for positive attitudes (80.40%) was markedly higher than that for negative attitudes (66.60%) (Table 2).

**Table 2:** Scores for artificial intelligence literacy, anxiety, and attitude.

Variable/Dimension	Mean ± SD	Minimum	Maximum	Scoring rate (%)	Total score range	Rank
AILS						
Total score	5.27 ± 0.90	2	7	75.29	1–7	–
Awareness	5.37 ± 1.10	1	7	76.71		2
Usage	5.15 ± 1.10	1	7	73.57		3
Evaluation	5.40 ± 1.24	1	7	77.14		1
Ethics	5.13 ± 1.05	2	7	73.29		4
AIAS						
Total score	3.59 ± 0.57	1	7	51.29	1–7	–
Learning	3.20 ± 1.73	1	7	45.71		4
Job replacement	3.86 ± 1.74	1	7	55.14		2
AI configuration	3.60 ± 1.86	1	7	51.43		3
Socio-technical blindness	3.95 ± 1.80	1	7	56.43		1
GAAIS						
Total score	3.70 ± 0.65	1	5	74.00	1–5	–
Positive	4.02 ± 0.77	1	5	80.40		1
Negative	3.33 ± 1.06	1	5	66.60		2

Scoring rate = Mean score/Total score × 100%.

AILS, Artificial Intelligence Literacy Scale; AIAS, Artificial Intelligence Anxiety Scale; GAAIS, General Attitudes Toward Artificial Intelligence Scale.

### 3.3 Factors influencing AI literacy, anxiety, and attitudes among registered nurses

As shown in Table 3, several demographic characteristics revealed significant differences regarding the AI literacy, anxiety, and attitudes of registered nurses. Regarding the artificial intelligence literacy of registered nurses, higher literacy levels were observed among those with higher educational attainment (*p* = 0.001) and shorter length of employment (*p* = 0.005). Nurses attending artificial intelligence training an average of 4–5 times per year demonstrated the highest literacy levels, whereas those attending ≤3 times exhibited the lowest (*p* = 0.003). Furthermore, nurses from tertiary hospitals had significantly higher artificial intelligence literacy levels than those from community hospitals (*p* < 0.001). Higher literacy levels were also identified in nurses whose departments were equipped with AI-related devices, those who had previously attended artificial intelligence training, and those who utilized electronic devices with higher proficiency and frequency (*p* < 0.001). Gender, age, and professional title showed no statistically significant differences (*p* > 0.05). In terms of artificial intelligence anxiety, nurses with 11–20 years of employment reported the highest anxiety levels, whereas those with ≤10 years reported the lowest (*p* = 0.004). Lower artificial intelligence anxiety was observed among nurses whose departments were equipped with AI-related devices (*p* = 0.013), as well as those from higher-tier hospitals, those with greater proficiency and higher frequency of electronic device use, and those who had attended artificial intelligence training (*p* < 0.001). Conversely, gender, age, educational level, professional title, and the average number of training sessions per year had no statistically significant effects (*p* > 0.05). Regarding nurses’ attitudes toward artificial intelligence, male nurses demonstrated significantly more positive attitudes compared to female nurses (*p* = 0.034). More positive attitudes toward artificial intelligence were also significantly associated with higher hospital tiers, greater proficiency and frequency of electronic device use, departmental availability of AI-related devices, and previous participation in artificial intelligence training (*p* < 0.001). Age, educational level, length of employment, professional title, and the average number of training sessions per year were not statistically significant (*p* > 0.05).

**Table 3:** Univariate analysis of artificial intelligence literacy, anxiety, and attitude.

Basic characteristics	Groups	AILS			AIAS			GAAIS		
		Median (Q_1,_ Q_3_)	*Z/H*	*P*	Median (Q_1,_ Q_3_)	*Z/H*	*P*	Median (Q_1,_ Q_3_)	*Z/H*	*P*
Gender	Male	5.38 (4.50, 6.00)	−0.242	0.809	3.45 (1.49, 4.87)	0.794	0.427	3.80 (3.22, 4.47)	−2.123	0.034
	Female	5.33 (4.58, 5.92)			3.71 (2.43, 4.67)			3.53 (3.13, 4.20)		
Age	≤30	5.42 (4.50, 6.00)	3.937	0.140	3.71 (2.19, 4.57)	2.869	0.238	3.63 (3.07, 4.20)	1.221	0.543
	31–40	5.33 (4.58, 5.92)			3.71 (2.43, 4.69)			3.47 (3.13, 4.20)		
	>40	5.17 (4.50, 5.83)			3.76 (2.62, 4.74)			3.67 (3.13, 4.33)		
Education	Secondary specialized school	4.33 (3.79, 5.40)	15.813	0.001	4.81 (3.37, 5.12)	5.628	0.131	3.13 (3.02, 3.67)	7.383	0.061
	Junior college	5.25 (4.50, 5.92)			3.90 (2.43, 4.71)			3.53 (3.07, 4.13)		
	Bachelor’s degree	5.34 (4.58, 5.92)			3.71 (2.38, 4.67)			3.60 (3.13, 4.27)		
	Master’s degree or higher	5.67 (5.42, 6.46)			2.62 (1.62, 4.08)			4.30 (3.23, 4.73)		
Years of experience	≤10 years	5.42 (4.58, 6.00)	10.636	0.005	3.55 (2.10, 4.38)	11.147	0.004	3.67 (3.13, 4.27)	2.242	0.326
	11–20 years	5.29 (4.58, 5.92)			3.90 (2.57, 4.81)			3.47 (3.13, 4.20)		
	>20 years	5.08 (4.42, 5.67)			3.86 (2.58, 4.77)			3.67 (3.13, 4.33)		
Hospital tier	Community hospital	4.75 (4.17, 5.50)	34.558	<0.001	4.00 (3.08, 5.00)	16.824	<0.001	3.33 (3.00, 3.87)	18.813	<0.001
	Regional hospital	5.33 (4.58, 6.00)			3.76 (2.33, 4.81)			3.47 (3.13, 4.13)		
	Tertiary care hospital	5.42 (4.58, 5.92)			3.57 (2.29, 4.52)			3.67 (3.13, 4.33)		
Professional title	Nurse	5.33 (4.42, 6.00)	1.576	0.813	3.62 (2.14, 4.52)	1.714	0.788	3.60 (3.07, 4.20)	7.594	0.108
	Senior Nurse	5.33 (4.50, 5.92)			3.81 (2.24, 4.71)			3.53 (3.07, 4.13)		
	Nurse-in-Charge	5.29 (4.58, 5.92)			3.71 (2.48, 4.71)			3.53 (3.13, 4.20)		
	Associate Chief Nurse	5.17 (4.54, 5.83)			3.62 (2.33, 4.60)			3.67 (3.13, 4.37)		
	Chief Nurse	5.50 (4.67, 6.04)			3.62 (2.79, 4.86)			4.27 (3.47, 4.53)		
AI equipment availability	Yes	5.50 (4.94, 6.25)	−6.358	<0.001	3.19 (1.92, 4.80)	2.497	0.013	3.73 (3.13, 4.40)	−4.007	<0.001
	No	5.17 (4.50, 5.83)			3.83 (2.57, 4.67)			3.53 (3.07, 4.13)		
Prior AI training	Yes	5.50 (5.00, 6.19)	−8.281	<0.001	3.38 (2.00, 4.62)	3.587	<0.001	3.80 (3.13, 4.47)	−6.186	<0.001
	No	5.08 (4.50, 5.67)			3.90 (2.65, 4.71)			3.47 (3.07, 4.07)		
Frequency of AI training (peer year)	≤3	5.50 (4.83, 6.08)	11.744	0.003	3.45 (2.14, 4.62)	3.794	0.150	3.73 (3.13, 4.40)	3.301	0.192
	4–5	5.63 (5.00, 6.44)			3.10 (2.00, 4.67)			3.87 (3.27, 4.47)		
	>5	5.58 (5.50, 6.50)			2.69 (1.00, 4.39)			4.03 (3.13, 4.78)		
Device proficiency	Very proficient	5.67 (5.08, 6.33)	177.905	<0.001	3.29 (1.86, 4.38)	40.332	<0.001	3.87 (3.13, 4.47)	57.718	<0.001
	Generally proficient	5.00 (4.50, 5.58)			4.00 (2.71, 4.76)			3.47 (3.07, 4.07)		
	Not proficient	4.54 (4.00, 5.25)			4.00 (3.77, 5.30)			3.13 (3.00, 3.68)		
Frequency of electronic device use	Often	5.75 (5.25, 6.42)	131.334	<0.001	3.00 (1.67, 4.20)	38.173	<0.001	3.97 (3.20, 4.47)	57.310	<0.001
	Occasionally	5.17 (4.50, 5.67)			3.90 (2.67, 4.71)			3.53 (3.13, 4.13)		
	Very few	4.92 (4.25, 5.67)			4.00 (2.71, 4.86)			3.27 (3.00, 4.00)		

Values are presented as Median (Q_1,_ Q_3_) due to non-normal distribution.

Mann–Whitney *U* test was used for two-group comparison; Kruskal-Wallis H test was used for multi-group comparison.

AILS, Artificial Intelligence Literacy Scale; AIAS, Artificial Intelligence Anxiety Scale; GAAIS, General Attitudes Toward Artificial Intelligence Scale.

Q_1_: first quartile; Q_3_: third quartile.

### 3.4 Correlation and linear regression analysis of AI literacy, anxiety, and attitude among registered nurses

The results of the correlation analysis, presented in Table 4, indicate that nurses’ AI literacy was negatively correlated with AI anxiety (*r* = −0.338, *p* < 0.001) and significantly positively correlated with AI attitude (*r* = 0.551, *p* < 0.001). Furthermore, a significant negative correlation was observed between AI anxiety and AI attitude (*r* = −0.541, *p* < 0.001). Table 5 presents the results of the subsequent regression analysis exploring the independent factors influencing registered nurses’ artificial intelligence anxiety and attitudes. The model assumption testing showed that the variance inflation factors (*VIF*) for all predictor variables ranged between 1.050 and 2.437, indicating the absence of significant multicollinearity. The standardized residual histograms exhibited a symmetrical distribution, and the points on the normal *P–P* plots closely aligned with the diagonal line, confirming that the residuals met the assumption of normality. Furthermore, the scatterplots of standardized residuals against standardized predicted values demonstrated a uniform distribution, with the vast majority of cases falling within the ±3 range, thereby satisfying the homoscedasticity requirement and indicating no severe outliers. The specific results of the regression analysis indicated that artificial intelligence literacy was an independent predictor of artificial intelligence anxiety (*B* = −0.453, *p* < 0.001), accounting for 9.9% of its variance. Similarly, artificial intelligence literacy was the strongest independent predictor of attitudes toward artificial intelligence (*B* = 0.377, *p* < 0.001), explaining 29.4% of the variance. Additionally, proficiency and frequency of electronic device use were significant predictors of artificial intelligence anxiety. Specifically, nurses who were not proficient in device operation (*B* = 0.489, *p* = 0.033) and those who used devices only occasionally (*B* = 0.246, *p* = 0.024) reported significantly higher artificial intelligence anxiety than those who were highly proficient and frequent users. Gender was found to be a significant independent factor influencing technological attitudes (*B* = 0.199, *p* = 0.011). Other covariates, including length of employment, hospital tier, departmental availability of AI-related devices, and previous participation in artificial intelligence training, did not reach statistical significance.

**Table 4:** Correlation analysis between artificial intelligence literacy, anxiety, and attitude.

Item	AILS	AIAS	GAAIS
AILS	–		
AIAS	−0.338**	–	
GAAIS	0.551**	−0.541**	–

^**^P < 0.001.

AILS, Artificial Intelligence Literacy Scale; AIAS, Artificial Intelligence Anxiety Scale; GAAIS: General Attitudes Toward Artificial Intelligence Scale.

**Table 5:** Multivariate regression analysis of anxiety and attitudes toward artificial intelligence.

Variable	AIAS						GAAIS					
	*B*	SE	*t*	*P*	95% CI	VIF	*B*	SE	*t*	*P*	95% CI	VIF
Constants	5.768	0.324	17.813	<0.001^**^	5.132, 6.403	–	1.742	0.114	15.254	<0.001^**^	1.518, 1.966	–
Gender	–	–	–	–	–	–	0.199	0.078	2.559	0.011^*^	0.047, 0.352	1.004
Years of experience (Ref: >20 years)												
≤10 years	−0.199	0.126	−1.571	0.116	−0.446, 0.049	2.437	–	–	–	–	–	–
10–20 years	0.048	0.125	0.380	0.704	−0.198,0.293	2.398	–	–	–	–	–	–
Hospital tier (Ref: Tertiary care hospital)												
Community hospital	0.236	0.123	1.916	0.056	−0.006, 0.478	1.078	−0.051	0.045	−1.129	0.259	−0.140, 0.038	1.075
Regional hospital	0.081	0.100	0.811	0.418	−0.115, 0.277	1.050	−0.057	0.037	−1.538	0.124	−0.129, 0.016	1.050
AI equipment availability	0.081	0.118	0.681	0.496	−0.151, 0.312	1.461	−0.031	0.043	−0.715	0.475	−0.116, 0.054	1.456
Prior AI training	−0.039	0.105	−0.370	0.711	−0.246, 0.168	1.532	0.051	0.039	1.321	0.187	−0.025, 0.127	1.528
Device proficiency (Ref: Very proficient)												
Generally proficient	0.040	0.091	0.442	0.658	−0.138, 0.219	1.246	0.023	0.033	0.702	0.482	−0.042, 0.089	1.232
Not proficient	0.489	0.229	2.136	0.033^*^	0.040, 0.939	1.177	−0.122	0.084	−1.441	0.150	−0.287, 0.044	1.175
Frequency of electronic device use (Ref: Often)												
Occasionally	0.246	0.109	2.265	0.024^*^	0.033, 0.459	1.838	−0.051	0.040	−1.277	0.202	−0.129, 0.027	1.834
Very few	0.220	0.130	1.696	0.090	−0.034, 0.474	2.036	−0.059	0.048	−1.244	0.214	−0.153, 0.034	2.035
AILS	−0.453	0.050	−9.123	<0.001^**^	−0.551, −0.356	1.247	0.377	0.018	20.650	<0.001^**^	0.341, 0.413	1.243

^*^P < 0.05, ^**^P < 0.001.

AIAS: *R*^2^ = 0.107, Adjusted *R*^2^ = 0.099, *F* = 14.969, *P* < 0.001; GAAIS: *R*^2^ = 0.299, Adjusted *R*^2^ = 0.294, *F* = 58.944, *P* < 0.001.

AILS, Artificial Intelligence Literacy Scale; AIAS, Artificial Intelligence Anxiety Scale; GAAIS, General Attitudes Toward Artificial Intelligence Scale.

*R*^2^, Coefficient of Determination; B, Unstandardized Regression Coefficient; SE, Standard Error; t, *t*-test for Significance; CI, Confidence Interval; VIF, Variance Inflation Factor.

## 4 Discussion

### 4.1 Current status of AI literacy, anxiety, and attitudes among registered nurses

This study demonstrates that the AI literacy level of registered nurses is in the upper-middle range, aligning with findings by Kahraman et al. (31). Regarding Turkish perioperative nurses and Migdadi et al. (31, 32). These results suggest that the current nursing workforce possesses a foundational knowledge base for applying AI in clinical practice. In this study, the artificial intelligence literacy evaluation and its awareness dimension yielded the highest scores, indicating that nurses are fully aware of the inevitable trend toward the integration and development of AI in nursing. Alruwaili et al. (33) noted that nurses generally acknowledge AI’s importance in enhancing care quality. Such evaluative capabilities and cognitive foundations can help nurses make informed decisions when navigating complex technical information, thereby facilitating the deep integration of technology and clinical practice (33). Conversely, scores in the usage and ethics dimensions were lower, highlighting a potential gap between theoretical knowledge and practical application. A study across three regions in China emphasized that if nurses’ practical application skills remain at the theoretical level, AI may become an expensive “laboratory ornament” rather than a clinical reality (34). Migdadi et al. (32) warned that AI applications lacking ethical awareness could lead to patient privacy breaches or bias in decision-making algorithms; indeed, the inherent algorithmic bias and data privacy leakage risks of artificial intelligence technology pose major challenges to current clinical applications. Because algorithm training often relies heavily on historical data, a lack of self-judgment awareness regarding specific diseases or demographic groups may lead to systemic misdiagnoses. Concurrently, the flow of medical big data can easily compromise patient privacy. These drawbacks often trigger a crisis of clinical trust among patients. Therefore, while strengthening technology implementation, nursing administrators should simultaneously establish and conduct relevant ethical training mechanisms (32). Concurrently, registered nurses exhibited a moderate level of anxiety regarding the clinical application of artificial intelligence technologies, which aligns with the findings of Ünal and Avcı et al. (17) in their study on neonatal nurses. This anxiety is not a rebellion against technology itself but rather a stress response to uncertainty. Maraş et al. (18) argue that nurse anxiety regarding robots and AI often stems from a lack of understanding of operational mechanisms. In this study, the dimension of socio-technical blindness anxiety yielded the highest score. This indicates that nurses’ primary concerns may center on the potential issues arising from the complex operational mechanisms of AI, as well as the loss of humanistic care in nursing resulting from the inability of AI algorithms to comprehend complex emotional interactions in clinical practice. Concurrently, the high level of job replacement anxiety reflects a sense of occupational crisis regarding being substituted by technology, alongside fears of social marginalization driven by an inability to keep pace with rapid socio-technical updates. This psychological state could potentially manifest as a source of resistance during the process of technology integration. Kahraman et al. (31) suggest that differentiated training based on individual backgrounds is necessary to help nurses perceive AI as a collaborator rather than a replacement. Notably, learning anxiety was relatively low, indicating that nurses are not inherently resistant to acquiring new skills. Demir-Kaymak et al. (35) noted that when individuals possess a certain knowledge base, anxiety regarding unknown technologies can be transformed into a motivation to learn. Nurses in this study maintained a generally positive attitude toward AI, with positive attitudes significantly outweighing negative ones. This optimistic tone is highly consistent with research by Abou Hashish and Alnajjar, reflecting nurses’ expectations for technological empowerment (36). Positive attitudes represent more than just psychological acceptance, Atalla et al. found that these attitudes correlate significantly with clinical reasoning and creative self-efficacy. When nurses view AI through a positive lens, they are more likely to treat it as a tool for cognitive enhancement, using it flexibly to solve complex clinical problems and improve efficiency (37). Özçevik Subaşı et al. (21) argued that attitudes serve as a critical moderating variable in technology integration, which may influence nurses’ tolerance levels when encountering technological errors. A positive attitude suggests that nurses are currently willing to allow the necessary time for adaptation and adjustment as these technologies are integrated into clinical practice.

### 4.2 Demographic differences in AI literacy, anxiety, and attitudes among registered nurses

Regression analysis revealed that after controlling for confounding factors, registered nurses’ artificial intelligence literacy was a negative independent predictor of anxiety, accounting for 9.9% of the variance in artificial intelligence anxiety. This finding aligns with the TAM 3.0 model, which posits that external antecedents can shape or modify an individual’s psychological anchoring beliefs. In this model, technological anxiety is conceptualized as a negative psychological anchor that hinders technology acceptance. The results of the regression analysis in the present study suggest that such anxiety is not an immutable or entrenched emotion; rather, nurses’ mastery of foundational artificial intelligence knowledge and skills may alleviate the fear associated with unfamiliar technologies (35). Migdadi et al. (32) also emphasized that when nurses possess a high level of AI ethical awareness, they are more perceptive to the boundaries of algorithmic bias and data privacy, thereby reducing the occupational insecurity associated with the “black box algorithm.” However, a mere increase in literacy does not fully alleviate anxiety; our results demonstrated that the strength of the association between literacy and anxiety was significantly weaker than that between literacy and attitudes. As reflected in the descriptive findings of anxiety levels, nurses’ anxiety regarding technology predominantly centers on socio-technical blindness and occupational replacement. This underscores that while objective knowledge can bolster nurses’ operational confidence, it may fail to eliminate deep-seated concerns regarding job substitution and the preservation of humanistic nursing care (32). Atalla et al. (37) pointed out that although a high level of AI literacy enhances application competence, if nurses perceive insufficient value in artificial intelligence technology, the accumulation of knowledge may conversely trigger panic regarding occupational and social marginalization, driven by the perceived impact of new technologies on traditional nursing modalities.

Secondly, as an independent positive predictor, literacy accounted for 29.4% of the variance in attitudes toward artificial intelligence. Within the framework of the Technology Acceptance Model (TAM), technological literacy, as an individual external characteristic, is regarded as a primary antecedent factor influencing users’ attitudes toward technology utilization. Although the mediating effects of variables such as perceived usefulness were not analyzed in the present study, the results of this regression analysis further confirm the critical importance of literacy as a baseline variable driving technology acceptance attitudes. Chen et al. (38) pointed out that nurses with high levels of literacy are more perceptive to the value of AI in assisting with diagnosis and improving the quality of nursing care, which positively enhances their attitudes through an increased trust in technology. Furthermore, Atalla et al. (37) pointed out that an enhancement in artificial intelligence literacy helps bolster nurses’ creative self-efficacy and clinical reasoning capabilities, enabling them to view artificial intelligence technology as a supportive tool for clinical practice rather than a burden. This sense of competence transforms the technology itself into an intrinsic driver for professional development, thereby fostering a more highly appreciative attitude toward its implementation (37, 39). Similarly, Zeng et al. (34) mentioned that this cognitively driven sense of competence serves as the very foundation for directly translating attitudes into an active intention to use the technology.

In addition to AI literacy, the regression analysis results also demonstrated that the proficiency and frequency of electronic device use were independent factors influencing registered nurses’ AI anxiety levels. Specifically, nurses who were more proficient and used electronic devices more frequently exhibited lower levels of AI anxiety. This finding aligns with the conclusions of Sommer et al. (40), who noted that prior digital experience serves as a foundation for nurses to learn new technologies, helping them establish a baseline psychological preparedness when facing complex algorithms. Erkayıran and Aslan (41) also pointed out that familiarity with daily electronic device use can translate into AI technology readiness; frequent exposure to digital tools in the workplace can mitigate the anxiety induced by unfamiliar technologies. Furthermore, univariate analysis revealed that nurses with a length of employment of 10 years or less (≤10 years) exhibited the highest levels of artificial intelligence literacy and the lowest levels of anxiety. This pattern—characterized by lower seniority being associated with higher literacy and lower anxiety—may be attributed to the fact that younger nurses typically bear a larger share of routine clinical duties, such as bedside nursing, documentation, and data reporting. The intense workplace demands may function as a motivational driver, prompting them to continuously learn new technologies in practice. Conversely, nurses with 11–20 years of employment reported the highest levels of artificial intelligence anxiety, consistent with the findings of Nirgiz et al. (42). Senior nurses may be more susceptible to entrenched mindsets during their long-term practice; as Amin et al. suggested, prolonged exposure to traditional clinical environments can lead to a degree of resistance among high-seniority nurses when confronted with AI-driven technological transformations (43). Moreover, this cohort is often situated at a critical crossroads of professional advancement and knowledge transition, enduring high workloads while simultaneously facing the pressure of technological updates, which collectively culminates in substantial anxiety. Additionally, the regression analysis indicated that gender was independently associated with registered nurses’ attitudes toward artificial intelligence, with male nurses demonstrating more positive attitudes than female nurses. In their investigation of the willingness to use generative artificial intelligence, Choe and Woo (44) similarly noted that male nurses tend to be more proactive in exploring emerging technologies. Nevertheless, because the gender distribution in the present study was highly imbalanced, this finding must be interpreted with caution.

In addition, univariate analysis revealed that nurses from higher-tier hospitals, those whose departments were equipped with AI-related devices, and those who had participated in relevant training displayed lower anxiety and more positive attitudes. Deng et al. (45) pointed out that nurses’ mastery of artificial intelligence technology is not merely the result of theoretical learning, but rather the combined effect of hands-on operational exposure and their surrounding environment. Higher-tier hospitals inject substantial resource investment and boast advanced informatics infrastructure, granting nurses more opportunities to interact with cutting-edge technologies. Conversely, because primary healthcare institutions face restricted resources and equipment, their nurses are long left on the periphery of technical learning, resulting in a pronounced digital divide. Tuncer et al. (46) emphasized in their study that nurses’ perception of a technology’s ease of use is a prerequisite for improving their attitudes toward emerging tools; moreover, the provision of a sufficient operational environment and technical support by hospitals constitutes an important condition for cultivating this perceived ease of use, whereas a lack of direct exposure can induce a fear of the unknown and negative emotions. Furthermore, Tseng et al. (47) mentioned that targeted educational training can enhance nurses’ information literacy and AI competency levels. However, when the regression model controlled for factors such as literacy and device-use behaviors, variables including hospital tier, the presence of AI equipment, and prior training lost their independent significance. This strongly reminds nursing administrators that simply chasing higher hospital rankings or mindlessly piling up hardware equipment is far from sufficient. While securing a baseline of physical equipment, greater focus must be placed on how to translate these objective resources into clinical nurses’ personal knowledge and literacy. This involves conducting targeted digital skills training, providing ample opportunities for clinical practice alongside technical support, and considering the formulation of technical usage regulations at an institutional level. Clearly defining responsibilities and principles of accountability will ultimately optimize nurses’ experience of technological ease of use and foster their trust in technology.

## 5 Strengths and limitations

The strengths of the present study lie in its multicenter design, utilizing a sample that spans registered nurses across tertiary, secondary, and community hospitals in Chongqing, stratified by hospital tier. This approach, to a certain extent, reflects the overall status of artificial intelligence literacy, anxiety, and attitudes among registered nurses across different institutional levels in this region. Furthermore, by employing regression analysis, this study elucidates the distinct impacts of literacy on anxiety and attitudes, thereby providing a theoretical foundation for developing targeted intervention strategies in the future. Nevertheless, several limitations must be acknowledged. Although a multicenter questionnaire survey was conducted, the study population was confined to the Chongqing region; consequently, the generalizability of the findings on a national scale remains limited. Secondly, the present study utilized stratified convenience sampling. Although stratification was performed based on hospital tiers, complete randomization was not achieved, which may introduce a potential selection bias in the enrollment of study participants. Furthermore, it is noteworthy that females accounted for 96.3% of the participants in this study, a distribution heavily influenced by the demographic profile of the nursing profession itself. Consequently, the interpretation of results regarding the gender variable must be treated with caution, as the findings may not fully reflect the true levels of AI literacy, anxiety, and attitudes among male registered nurses. Lastly, the present study adopted a cross-sectional descriptive design, which only allows for the elucidation of associations among registered nurses’ artificial intelligence literacy, anxiety, and attitudes, but precludes any causal inferences regarding these variables. Additionally, while general demographic data such as hospital tiers were analyzed, the potential influences of regional disparities, organizational management, and institutional technological environments were not fully accounted for. Future research could further expand the scope of relevant variables, utilize randomized sampling designs, and consider implementing large-sample longitudinal studies or intervention trials on a national scale. Such efforts would help rigorously map the specific causal pathways and directional mechanisms underlying registered nurses’ artificial intelligence literacy, anxiety, and attitudes.

## 6 Conclusion and implications

The survey revealed that registered nurses’ artificial intelligence literacy is at an upper-medium level, though notable deficiencies persist regarding its usage and ethical dimensions. While nurses generally exhibit a relatively positive attitude toward artificial intelligence technology, they concurrently experience a moderate level of anxiety. Specifically, this technological anxiety is most pronounced in the realms of socio-technical blindness and concerns regarding job replacement. Regression analysis indicated that enhancing literacy can mitigate registered nurses’ artificial intelligence anxiety and optimize their attitudes toward its integration. It is noteworthy, however, that while improved literacy bolsters nurses’ confidence in utilizing technology, it cannot entirely eradicate rational apprehensions stemming from inherent technological limitations, ethical uncertainties, and socio-technical marginalization. Furthermore, compared with objective external factors such as organizational environments or individual baseline demographics, nurses’ actual mastery and hands-on utilization of technological knowledge emerge as the critical determinants for successful clinical implementation. Consequently, future interventions should pivot from purely theoretical training toward integrated strategies that promote the synergistic development of technological competence and psychological resilience. In addition to providing external technical support, administrative efforts must focus on facilitating the practical application of AI in real-world clinical settings. Given the cross-sectional nature of this study, which precludes causal inferences, future longitudinal or intervention-based research is warranted to comprehensively validate the directional pathways and causal mechanisms underlying these variables.

## 7 Future research directions

Future research could employ qualitative methods, such as in-depth interviews, to explore the factors influencing registered nurses’ AI literacy, anxiety, and attitudes more comprehensively, addressing variables not covered in this study—such as socioeconomic status, specific organizational support policies, and department types. Given that the current study is a cross-sectional survey and cannot infer causality, longitudinal studies or intervention-based research should be conducted in the future to deeply validate the relationships between these variables. Additionally, large-scale randomized surveys could be carried out nationwide to enhance the representativeness and generalizability of the findings.

## Data Availability

The raw data supporting the conclusions of this article will be made available by the authors, without undue reservation.
