# Correction: Artificial intelligence literacy, anxiety, and attitudes among registered nurses across different hospital tiers: a multi-center cross-sectional study

**DOI:** 10.3389/fpubh.2026.1989985

**Published:** 2026-09-17

**Authors:** Yingqi Dai, Xili Zhao, Xiao Pan, Jiaxin Wei

**Affiliations:** 1 The Second Affiliated Hospital of Chongqing Medical University, Chongqing, China; 2 Acute and Critical Care Nursing Innovation and Translation Engineering Research Center of Chongqing Education Commission of China, Chongqing, China; 3 Chongqing Geriatric Specialized Nursing Clinical Diagnosis and Treatment Center, Chongqing, China

**Keywords:** anxiety, artificial intelligence, attitudes, literacy, registered nurses

Affiliations Acute and Critical Care Nursing Innovation and Translation Engineering Research Center of Chongqing Education Commission of China, Chongqing, China and Chongqing Geriatric Specialized Nursing Clinical Diagnosis and Treatment Center, Chongqing, China were omitted for author Xili Zhao. These affiliations have now been added for author Xili Zhao.

The original version of this article has been updated.

